# Age-Related Loss of Muscle Mass and Strength

**DOI:** 10.1155/2012/158279

**Published:** 2012-03-08

**Authors:** Geoffrey Goldspink

**Affiliations:** Departments of Surgery, Anatomy and Developmental Biology, University College Medical School, Royal Free Hospital Campus, Hampstead, London NW3 2PF, UK

## Abstract

Age-related muscle wasting and increased frailty are major socioeconomic as well as medical problems. In the quest to extend quality of life it is important to increase the strength of elderly people sufficiently so they can carry out everyday tasks and to prevent them falling and breaking bones that are brittle due to osteoporosis. Muscles generate the mechanical strain that contributes to the maintenance of other musculoskeletal tissues, and a vicious circle is established as muscle loss results in bone loss and weakening of tendons. Molecular and proteomic approaches now provide strategies for preventing age-related muscle wasting. Here, attention is paid to the role of the GH/IGF-1 axis and the special role of the IGFI-Ec (mechano growth factor/MGF) which is derived from the IGF-I gene by alternative splicing. During aging MGF levels decline but when administered MGF activates the muscle satellite (stem) cells that “kick start” local muscle repair and induces hypertrophy.

## 1. Introduction

When our hominid species evolved from several millions of years ago, ancient man was a hunter-gatherer, and survival required covering long distances. As well as stamina, homosapiens had to have sufficient strength to kill large animals for food. Ancient man would have sustained muscle injuries during hunting and tribal confrontations, and, from a Darwinian viewpoint, natural selection would have resulted in generations of offspring with strong and adaptable musculature; this includes rapid and effective tissue repair as this was also a requisite for survival and the continuation of the species. However, over most of this time the average life expectancy for most homosapiens was only about 25 years, that is to say a little beyond the age of reproduction. For example, in ancient Egypt the average life span was 24 years but now with developments in science and medicine this has increased by over 3-fold which presents problems for human society. In the more affluence society of today there are other factors such as overconsumption of food and alcohol and the failure to maintain an active, healthy life style. In Scandinavian countries family doctors prescribe exercise to improve the general fitness which enables individuals to maintain an active life style and to live longer. Longevity and the increasing percentage of elderly in the populations in many developed countries including the USA, Europe, and Japan present its own major socioeconomic as well as medical care problems. Therefore maintaining independence has now to be very much focused on the aging processes of the musculoskeletal system. 

Mechanical tissues are designed to respond to mechanical forces, and it is important to determine why there is a decreasing sensitivity of the transduction of mechanical signals that maintain muscles and to what extent this is due to inactivity or intrinsic tissue changes as we get older. These are not simple questions to answer as such factors as neurological input, blood flow, and fatigue resistance that include tissues other than muscle may become limiting factors. From the prospective of the author the information in this paper concentrates on that acquired over the last decade on changes at the cellular and molecular levels in aging muscle tissue as present day molecular genetics and proteomics methods have provided us with tools for studying the age-related muscle growth, adaptation, and repair. Sarcopenia is the term that is often used to describe the syndrome of age-related muscle loss which is somewhat unfortunate as this implies that it is a disease rather than an attenuation of processes that develop and maintain muscle in young healthy people. Postnatal growth of muscle is very much influenced by hormones which include growth factors and androgens, the circulating levels of which decrease with age. This decrease in hormone levels in the elderly has sometimes been referred to as the “somatopause” as this occurs in males and females. Supplementing the levels of these hormones has been found to be beneficial, for example, oestrogen and progesterone replacement therapy in women and administration of testosterone in elderly men to improve muscle strength. 

The insulin-like growth factor (IGF-I) system is beginning to receive considerable attention as it is involved in tissue growth, maintenance, and repair. Interestingly, an IGF gene is present in invertebrate animals. This and its receptor gene have been studied in the nematode worm *C. elegans * [1] as it is involved in determining the life span of the worm by suppressing cell death (apoptosis). Experiments have shown that the IGF gene and its receptor gene represent a primitive system involved in maintaining terminally differentiated cells. In this way these determine lifespan in the nematode worm [2] and have become a model for studying aging at the very basic level. The lifespan of vertebrates including man is of course much longer than the nematode worm. In higher animals the IGF-I system is similar but more sophisticated in that the family of genes and the alternate splicing of genes in vertebrates result in a number of gene products. In vertebrates during aging, muscles decline in strength and adaptability [3]. Coincidentally, levels of insulin-like factors decline. These are controlled by growth hormone (GH) produced by the pituitary gland and referred to as the GH/IGF-I axis which controls body mass particularly muscle mass. However, space does not permit a discussion of the combined effects of growth hormone and androgens on muscle during aging although this topic is still receiving much attention and not reviewed here except where they might be involved in GH/IGF-I axis [4].

However, this paper will concentrate mainly on the adaptation mechanism(s) that are linked to the repair processes via which myofibers adapt and/or are repaired after being subjected to mechanical strain. As skeletal muscle is a “postmitotic tissue” and cell replacement is not a means of repairing damage as it is in most tissues and there has to be a somewhat different but effective mechanism, otherwise the cellular units would undergo cell death and not be replaced. This does happen to some extent in advanced old age when the maintenance and reinnervation of the muscle fibers begins to fail. In normal muscle events associated with local damage and repair result in adaptation whereby the muscles increase in strength but this decreases with age. In diseases such as the muscular dystrophies muscle repair is not initiated in the normal way as certain cytoskeletal assemblies are defective or missing as in Duchenne muscular dystrophy that is the most severe type. In the mdx dystrophic mouse that is an animal model for human Duchenne muscular dystrophy, the muscles are unable to respond to mechanical stimuli by initiating the repair signalling including splicing the IGF-I gene towards the IGF-IEc. Because of the original confusion of what was called IGF-IEb which has almost the same sequence of IGF-IEc in the human and because its expression increased in response to mechanical signals, the latter was called mechano growth factor (MGF) [5]. In experiments in which the dystrophin complex has been restored by cell transfer, the appropriate signalling including the production of MGF is restored [6]. During normal ageing this mechanotransduction system apparently becomes increasingly less sensitive as the fibers become less compliance due to increased amount and stiffness of the fibrous connective tissue in the muscles. In addition to muscle stiffness, another factor is the drop in circulating levels of some hormones which influence the expression of the genes that result in the local signals that are produced in response to physical activity. Studies of cellular changes associated with age-related muscle atrophy indicated that there is a considerable loss of muscle fibers, motoneurons, and motor units [7]. In a more recent project studied in older men aged just over 70, with a follow-up study on the cohort when they approached 80 years of age, the muscle cross-sectional area and specific strength of the knee extensors had significant decreased [8]. Interestingly, there was some increase in the size of the type IIA fibers indicating that the recruitment pattern of the myofiber types changed, and this tended to compensate for the loss of fatigue resistance and strength. This paper will, however, concentrate on molecular biology and proteomic studies. By using these approaches we can begin to understand the atrophy processes associated with aging and the possibility of intervening in these processes of loss of muscle mass and strength, as we age.

## 2. Approaches to Treating Age-Related Muscle Loss

### 2.1. Treating the Somatopause

One of the changes during aging is that the circulating levels of GH drop markedly with age and one approach to counteract the effects of aging might have been to administer human GH as a recombinant peptide. In experiments with the author's group in collaboration with Professor Kjaer's group in Copenhagen, it was found that strength improved with the administration of growth hormone which apparently increases the level of the primary IGF-I transcript and there was a correlation between increased MGF levels and muscle mass measured by MRI scanning [9, 10]. More recently our group has been involved in detecting the misuse of GH by athletes but it should be appreciated that young healthy individuals are not GH deficient. Therefore, the response is likely to be different to that in older muscle tissue, particularly in those individuals in whom the circulating levels are well below that of their age group. A study by Yamaguchi et al. [11] showed that in hypophysectomized rat muscle the expression of IGF-I splice variants was more influenced by mechanical factors than endocrine status. This reaffirmed a earlier study carried out a good number of years ago by Goldberg [12] who used hypophysectomized rats and showed when muscles are under mechanical strain they still underwent hypertrophy indicating that regulation of muscle mass and strength are regulated at the muscle cellular level. Systemic administration has associated problems as GH is produced episodically, and there is a negative feedback so that administration of a large bolus of recombinant human growth hormone (rhGH) shuts down its endogenous secretion by the pituitary which produces the opposite result. Also elevated GH levels are associated with an increased in IGF-I and increased cancer risk as IGF-I is carcinogenic at abnormally high levels although it has been reported as repressing protein breakdown [13]. A higher risk of cancer is seen in acromegalic patients whose pituitary gland is overactive. These people are invariably tall and well muscled but have a high risk of cancer resulting from the higher circulating levels IGF-1. It is now becoming apparent that this may be a problem in young athletes and bodybuilders and the antidoping agencies are becoming concerned about this form of abuse. Hence there are considerable risks associated with elevating the serum GH levels beyond that which is normal for a particular age group artificial elevation of GH or IGF. The levels of several hormones are known to drop markedly with age including testosterone as well as GH that are involved in the maintenance of muscle mass. It is not likely that these would be used as a general treatment for age-related muscle loss although it may be indicated for some elderly individuals but this has to be combined with exercise to obtain an increase in strength [10]. As the endocrine system is complex and the chances of inducing unwanted side effects are reasons why the pharmaceutical industry is attempting to develop SARMS, these compounds stimulate specific androgen receptors, in particular the testosterone receptor, and already they have found their way into doping in certain sporting events.

### 2.2. Manipulation of the Balance between Protein Synthesis/Breakdown

The size of tissues in the body is determined mainly by a balance between the rate at which proteins are synthesised and the rate they are broken down. This is a way cells adapt to change in local environmental conditions and for removing nonfunctional, damaged proteins. Every few days approximately half the enzyme molecules within our body will be broken down and replaced. The turnover of the contractile proteins of muscle such as actin and myosin takes a little longer with about half of being replaced every two weeks or so. 

Using nonradioactive isotope methods it was found that as we get older our muscle proteins are degraded and rebuilt at less frequent intervals but still the process is very dynamic. Protein degradation requires energy as does the synthesis of muscle proteins, and it appears that the balance is more towards decreased protein synthesis except in some disease states, for example, endotoxin poisoning, in which muscle protein breakdown can be very rapid. Any proposal for increasing muscle mass during aging by slowing the degradation process during healthy aging would seem to be physiologically undesirable as continual replacement of proteins is particularly important in a mechanical tissue as this is the way of ensuring there is no build-up of nonfunctional proteins. Also in tissues such as muscle there would probably be a reduction in specific strength as increased mass does not necessarily mean an increased ability to generate muscle force, and there is no rationale for increasing body mass without commensurate increases in strength.

### 2.3. Blocking Negative Regulation of Muscle Mass

There has been a lot of interest in a negative muscle regulatory factor that has been named myostatin. As the name implies this factor reduces muscle growth, and knocking this out therapeutically offered prospects of increasing muscle mass and strength. The myostatin gene, when mutated, is responsible for “double muscling” in certain breeds of cattle, such as the Belgian Blue, which exhibit very considerable muscle hypertrophy [14] and also in a few human subjects in which the gene is mutated [15]. However, McMahon et al. [16] reported that in the myostatin knockout mouse, the lack of myostatin did not ameliorate disuse atrophy. As discussed below skeletal muscle is a postmitotic tissue and in order to increase in size or to repair the myofibers have to obtain extra nuclei. Postnatal inactivation of the myostatin by gene targeting results in myostatin “knock out” with an increased number of satellite cells [17], and an increase in muscle mass reverses the quiescence state of these muscle progenitor cells [18]. However, it was shown in these mice [19] that the increased muscle mass resulted in decreased specific contractile force as there was apparently an accumulation of nonfunctional protein. The suggested therapy for muscle loss that entails partially knocking out/knocking down myostatin is also probably not a good strategy as this is associated with a loss of oxidative capacity of the muscles as well as impaired respiratory and cardiovascular function [20]. In one young boy who has a myostatin double allele knockout, he has increased muscle mass and strength and this would obviously be an advantage in certain sports, but to be “muscle bound” is not necessarily an advantage in many activities that require power rather than maximum force nor would it be desirable for older people. Interestingly, a study on aging weight lifters did show that myostatin levels decrease when they resume exercise so this subject needs further investigation. Clearly if the use of an antimyostatin strategy results in an increase of mass without a commensurate increase in strength this would handicap rather than help elderly individuals.

### 2.4. Augmenting the GH/IGF-I System for Maintaining Muscle Mass and Strength

Even in individuals who exercise regularly, the ability to maintain muscle mass and strength diminishes with age, and this is associated with the marked drop in the circulating serum levels of IGF-I. Muscle hypertrophy and wasting have been studied at the gene level based on the realisation that there must be local as well as systemic regulators of muscle growth. Approximately 15 years ago the author's group cloned a factor that proved to be involved in local regulation of muscle mass and which is derived from the splicing of the IGF-I gene. To do this we used an animal model in which we could make muscle grow rapidly. Previous work had shown that the tibialis anterior in the mature rabbit whilst held in the stretched position by a plaster cast and when electrically stimulated using an implanted microcircuit increased in mass by 35% in just over 7 days [21]. It was known that muscles adapt to an increased functional length by adding sarcomeres in series at the ends of the existing myofibrils, and if they are also subjected to electrical stimulation they also increased in girth and also added more sarcomeres in parallel as well as in series. RNA was extracted from these muscles that were undergoing rapid growth and using differential display, and we detected a RNA transcript that is expressed in stretched/exercised but not in resting muscles. This mRNA was converted to cDNA, sequenced, and referred to the genome data base which showed it to be derived from the insulin-like growth factor (IGF-I) gene. However, its 3′ sequence was different to the liver or systemic type of IGF-I (IGF-IEa) [22]. 

**Figure 1:**
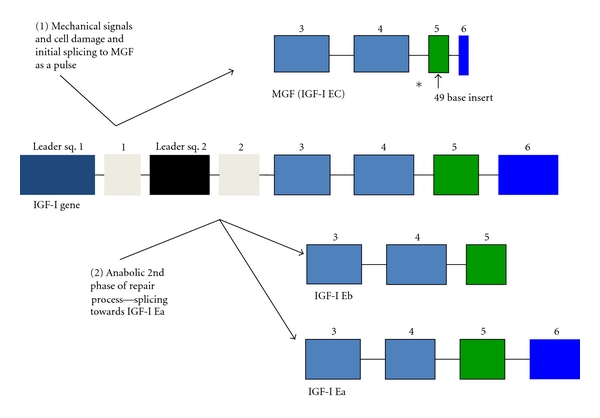
Schematic representation of the splicing of the human IGF-I gene in human muscle to produce human MGF (IGF-1Ec) as well as IGF-IEa and IGF-IEb. The IGF-I gene has two start sites located in or near exons 1 and 2. In response to a exercise/damage the IGF-I gene is first spliced to produce MGF. This involves a reading frame shift that results in the unique carboxy terminal sequence (E domain) of MGF (IGF-I Ec). The expression of the IGF-I gene is initiated by certain hormones, and this becomes a problem as during aging GH and testosterone levels decrease resulting in decreasing levels of the primary transcript so less can be spliced to MGF when the muscle is mechanically challenged. Also it appears that the mechanotransduction and signalling system that is upstream of the activation of the IGF-I gene also become less sensitive, and less MGF is produced.

Later work showed that in human muscle 3 main types of IGF-I can be spliced from the IGF-I gene (Figure 1), a systemic (liver) type of IGF-I (IGF-IEa), IGF-I Eb, that is not the same as IGF-IEb in rodents and the newly discovered splice variant IGF-IEc. As the terminology of the IGF-Is is a problem when attempting to apply it to nonhepatic tissues and different species we called this newly discovered splice variant mechano growth factor (MGF) as it is expressed in response to mechanical stimuli and muscle damage. 

As well as MGF expression was mechano sensitive its 3′ sequence was unique and encoded a different E domain at the carboxy end (Figure 1) that presumably has several distinct actions. The reason the C terminal end of MGF is different to the other IGF-I splice variants is because during the splicing of the IGF-I gene at exon 5 has 52 bases in the human (49 bases in rabbit and rat) and this introduces “a reading frame shift” at the 3′ end. Following the initial splicing to MGF (human IGF-IEc) which has been found to initiate muscle growth and repair, the IGF-I gene is later switched to IGF-IEa which is a main anabolic agent and some studies suggest that it initiates the fusion of satellite cells with the myofibers and the expression of myogenic genes [22–24]. MGF, however, appears to have a special role in muscle repair that involves its unique E domain sequence that arises from this reading frame shift [25]. As well as activating and replenishing the muscle stem/precursor cell pool (see Figure 1) this unique E domain peptide has also been found to have several roles in limiting tissue damage but space does not permit these to be reviewed here. There is good evidence that the age-related changes in muscle mass and strength result from a decreasing ability to produce MGF [26–28], and this seems likely to be the case in other tissues. From a physiological point of view this is interesting as the age-related decline in the ability to respond to physical signals by producing MGF is presumably related to a dulling of the mechanotransduction system which is still not understood.

### 2.5. Exercise and Decreasing Ability to Respond to Active Muscle Stretch during Aging

**Figure 2:**
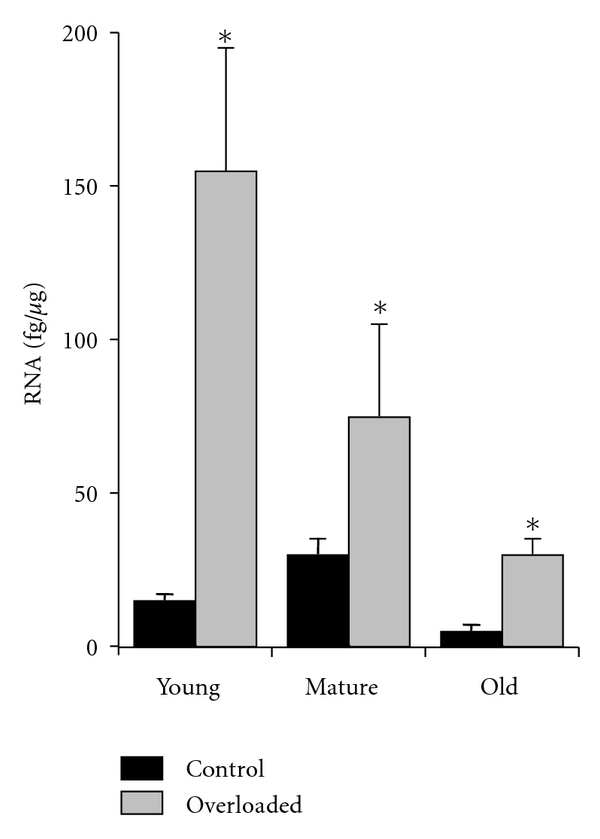
The ability to express MGF in response to mechanical strain with age. Animal experiments overloading the rat soleus and plantaris muscles by cutting the gastrocnemius muscle of rats from 3 age groups. In the young rat the overloaded muscles produced high levels of MGF but in the older rats the induced levels were much lower even though these rats were much heavier. The middle-age rats the MGF levels were intermediate between the young and the old in response to mechanical overload, according to Owino et al. [27].

**Figure 3:**
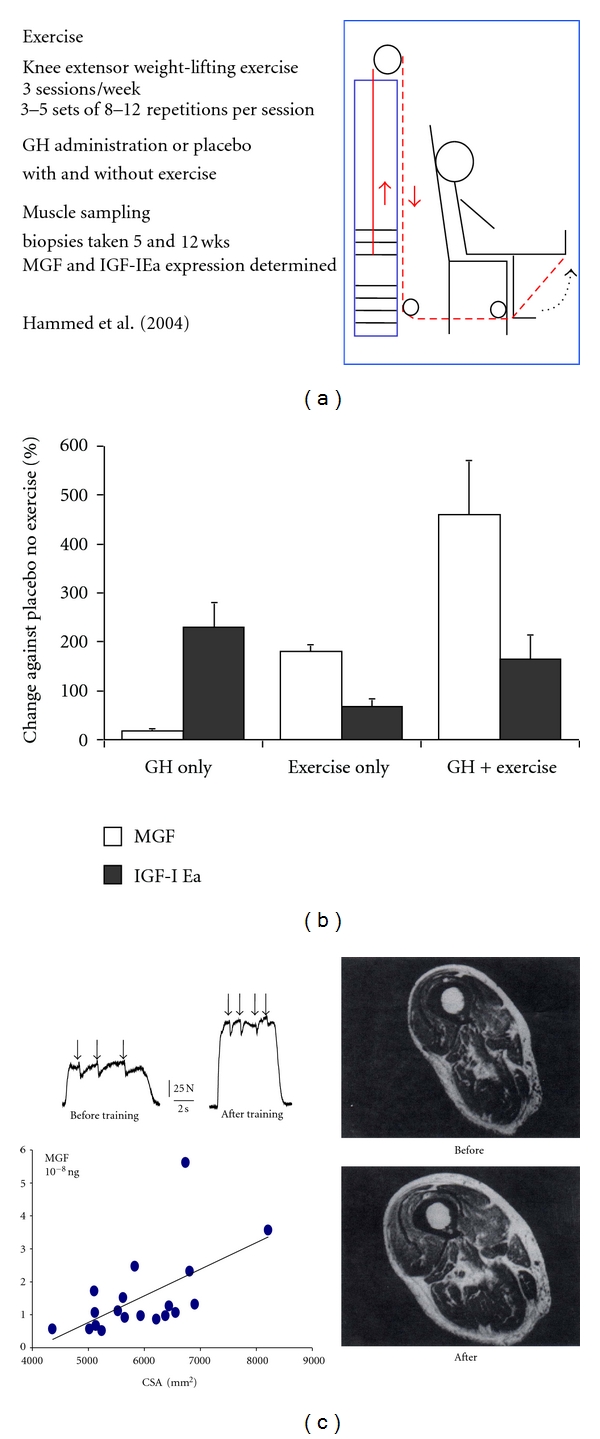
(a) Exercise data in which muscle MGF levels were determined in elderly men; some receiving exercise only and another group exercise with GH and in which biopsies were taken and MGF levels were measured and compared with muscle cross-sectional area and strength. (b) shows the relationship between the induced levels of MGF and the increased strength in the experiments presented in (a). (c) presents on the left side the change in muscle cross-sectional area after exercise combined with GH treatment and increased MGF expression in elderly men. On the right are two scans showing the difference when exercise is combined (top right) or not combined with GH treatment (bottom left). See Hameed et al. [28].

As shown in the rat (Figure 2) and human subjects a major intrinsic effect is increased load, but this response becomes dulled during aging [26]. Also in studies on elderly human subjects [27] this was found to be related to decreased MGF and IGF-1Ea which could be improved by administration of GH which increases the expression of the IGF-I gene. (see Figures 3(a)–3(c)). As mentioned above in Section 2.4 we understand more about the endocrine aspects than the physical signalling and signalling molecules involved in muscle adaptation involved in maintaining and repairing our muscles as we age. 

The detection of mechanical strain is thought to involve focal adhesion kinases (FAKs) [30] which are activated by mechanotransducers systems that link the very long titin molecules that run through the myofibrils to the tendons. These also connect in 3 dimensions as the myofibrillar system is linked to the surrounding basal lamina and response elements, and when these are missing, for example, dystrophin or defective is responsible for the muscle wasting conditions. The connective tissue of the tendons and ligaments also transmits the forces generated by sarcomeres of the muscles to tendons, bones, and ligaments. These mechanical forces generated by skeletal musculature are thus also involved in maintaining the whole of the musculoskeletal system. It seems that as we grow older this mechanotransduction system becomes less sensitive because of the decreased compliance of the connective tissue due to cross-linking of the collagen which seems to occur in all tissues. In some experiments on mice subjected to repeated exercise the author's group found that regular exercise improved muscle compliance during aging but it was still not as good as in young mouse muscles [30]. These experiments preceded the discovery of MGF which now offers good prospects for its use as a therapeutic compound for treating age-related muscle loss as well as muscle cachexia in a range of diseases although there seems to be more interest in its use as a doping agent as it is available over the internet and it is now being produced using recombinant *E. coli* methods, and therefore it will become relatively inexpensive. Unfortunately, during aging the muscles become less compliant and become less able to produce MGF [26–28, 31]. As with other tissues muscles become less compliant which apparently occurs due to cross-linking in the connective tissues and possibly other factors, and in some long running mouse experiments we showed that regular exercise improved muscle compliance during aging but it was still not as good as in young mouse muscles [30]. Also in diseases such as the muscular dystrophies, the impairment is often the absence or incorrect conformation of the certain linking molecules such as dystrophin that apparently results from an inability to produce MGF. From a physiological point of view this is of considerable interest as the initiation of the activation of the IGF-I gene and the switch in splicing to produce MGF must involve a mechanotransduction system. The detection of mechanical strain is thought to involve focal adhesion kinases (FAKs) which change conformation when stretched which phosphorylate other signal molecules [32]. The impairment of mechanosignalling appears to be defective in diseases such as the muscular dystrophies in which splicing of the IGF-I gene is deficient but transfer of normal mesenchymal stem cells into the dystrophic mouse restored its ability to produce MGF [33]. Therefore, there are probably good prospects for use of therapeutic compounds such as MGF for treating age-related muscle loss as well as muscle cachexia in a range of diseases. Unfortunately, at the present there seems to be more interest in its use as a doping agent as it is available over the internet and it is now being produced using recombinant *E. coli* methods, and therefore it can become relatively inexpensive. 

### 2.6. Muscle Progenitor/Satellite Cells in Muscle Repair, Adaptation, and Maintenance

Skeletal muscle and most neurological tissues are postmitotic tissues, and age-related deficits require a somewhat different understanding to that of other tissues. The term “postmitotic tissue” is used as after embryonic tissue is complete, the myofibers have residual myoblasts in the space between the plasma membrane and the basal lamina. Because of their position these mononucleated cells were originally called satellite cells. These upon the appropriate signal undergo proliferation and one of the progeny fuses with the muscle fibres, and the other mononucleated muscle stem cell enters quiescence. 

It has been realized for some time that these cells provide the extra nuclei for postnatal growth and regeneration [32] and in response to mechanical strain and local injury [32–38]. Growing up mononucleated myoblasts for injection into muscles of patients has been the goal for several groups but as most of these die after injection, this does not seem preferable to introducing MGF as a stabilized peptide or gene construct as one injection provides many additional myofiber nuclei. The initial splicing of the IGF-I gene to produce MGF is within the first few days after the mechanical challenge and coincides with the activation of the muscle stem cell pool [36–38]. Indeed, the initiation of muscle stem cells resulting from exercise has been shown in human muscle after a single bout [37]. Bamman's group [38] found that after resistance exercise there was a correlation between cyclin D1 activity with MGF expression demonstrating cell replication in this postmitotic tissue. In another study it was shown that a decrease in myostatin levels results in an increase in specific strength so it is assumed that muscle satellite (stem) cell activation is positively regulated by MGF and not by removal of myostatin, the negative regulator [18]. Following the expression of the IGF-I gene the satellite cell pool undergoes periods of replenishment lasting just a few days [34], and the initial splicing to MGF expression following a mechanical challenge fits with the timing of the expansion of the muscle stem (satellite) numbers [30, 39]. Both MGF and IGF-IEa are apparently involved during muscle hypertrophy and repair as this involves replenishing the muscle satellite (stem) cell pool which “kick starts” the growth, and repair processes the anabolic response to IGF-I Ea. Stem cell therapy has been proposed for extending the life and the quality of life but in the case of muscle loss and frailty and in muscle cachexia in a range of diseases. However, transplanting muscle cells would not seem to be necessary if the existing stem cells can be induced to multiply by merely administering MGF as a stabilised peptide or by gene therapy. 

### 2.7. Activation of Muscle Progenitor Cells in Muscle Wasting and during Aging

**Figure 4:**
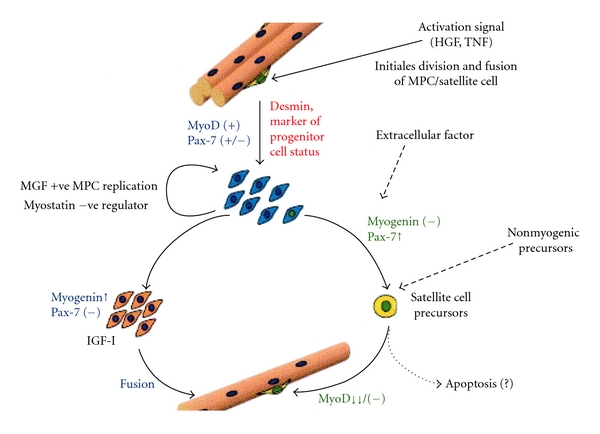
Illustrates role of the MGF C terminal peptide in activating the replication of the mononucleated myoblasts (otherwise termed as satellite cells or muscle progenitor/stem cells). These are the source of the extra nuclei required for local muscle repair. MGF in young normal muscle replenishes the pool of stem cells, and myostatin acts as a negative regulator and pushes then into a quiescent mode. Following the activation of the mononucleated myoblasts to undergo mitosis one of the pair will fuse with the myofiber and switch into myogenic gene expression for the production of contractile such as actin and myosin and other muscle proteins.

**Figure 5:**
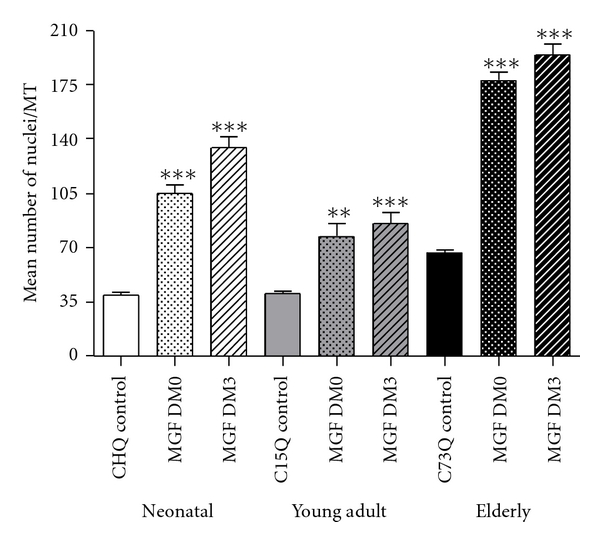
In elderly muscle MGF treatment activates “resting” cells to proliferate and fuse to donate nuclei. Cultures from neonatal, young adult, and old adult muscle were treated with the unique MGF peptide (MGF E) 24aa at a concentration of 100 ng/mL at day 0 or day 3 of differentiation. Immunofluorescence was performed on cultures with an antibody directed against Desmin (a marker of progenitor cell status). In elderly muscle there are still some muscle satellite/stem cells in a quiescent state but these are not normally activated because older muscle does not normally produce much MGF. However when the unique E peptide of the MGF was added to cultures from elderly muscle these cells replicated. The prospects of “awakening” of these resting progenitor cells appear to offer a means of enhancing muscle maintenance and repair in the elderly offers and in certain diseases such as muscular dystrophy and ALS (Kandalla et al. [29]).

It has been shown that age-related sarcopenia is related to IGF-I signaling [34, 35] and in particular reduced ability of muscle to express MGF, a splice variant of the IGF-I gene and also IGF-IEa which is the main anabolic agent or increased muscle protein content [34, 36]. This has been associated with muscle aging in animals [22, 26] and humans [27, 28], and because of this, the need to activate the muscle stem/progenitor cells to increase muscle mass has been highlighted in a number of publications [35–41]. For this purpose the unique E peptide sequence of MGF (MGF-24aa-E) was used to study the activation of the human satellite cells (mononucleated myoblasts) taken from patients with muscle wasting diseases including two muscular dystrophies and ALS [41]. If IGF-I was added together with the MGF peptide then it became apparent that the mononucleated cells went through the next stage of activation and fused with the muscle myofibers in the cultures. Therefore, it seems that both the unique MGF E domain peptide and IGF-I are required for the repair process. Work with Dr. Gillian Butler-Browne's group [42] shed more light on the repair process in relation to aging. With this group we investigated the actions of the MGF E-24aa peptide using differentiated cultures and analyzed to estimate (1) the fusion index, (2) the percentage of unfused (desmin positive) reserve cells, and (3) the mean number of nuclei per myotube. A fusion index was determined by counting the number of nuclei in the differentiated myotubes with more than two myonuclei as a percentage of the total number of nuclei (mononucleated and multinucleated). The percentage of unfused positive cells was calculated, by counting the number of unfused, desmin-positive-single-nucleus cells as a percentage of the total number of nuclei (1000 nuclei per dish in triplicate). To measure the extent of cellular proliferation within a culture, the incorporation of 5-bromo-2′-deoxyuridine (BrdU) was used. During the replicative life span, proliferating cells were incubated for 72 h in the presence of 10 *μ*g/mL of BrdU. To identify the cells that had incorporated BrdU, using a monoclonal antibody directed against BrdU, and the nuclei were counterstained with DAPI. It was found that this MGF peptide significantly increases the proliferative life span of satellite cells isolated from neonatal and young adult but not from old adult muscle. However, it was noted that the mean number of nuclei per myotube and the fusion index were higher in cultures from older subjects. Interestingly, hypertrophy was observed in satellite cell cultures from all three age groups. This was associated with a significant decrease in the percentage of “reserve” cells correlated with an increase in the number of nuclei in the myotubes that occurred when MGF-24aa-E peptide was added to cultures. It is concluded that only 24aa of the MGF isoform of IGF-1 has a marked ability to initiate and enhance satellite (progenitor) cell replication and fusion for muscle repair and maintenance. Aging muscle has a declining ability to produce MGF but it was found that administration of the MGF peptide prevents satellite cells entering senescent and by activating these reserve progenitor cells to fuse, and this provides the extra nuclei required for continued tissue maintenance. Unfortunately as mentioned above as we become older our ability to produce MGF declines along with GH and IGF-I. Although the older myoblasts cultures showed a dramatic decrease in the number of cycling cells following the addition of MGF-24aa-E, their ability to induce hypertrophy was assessed following a single dose of MGF-24aa-E which was given immediately or just after 3 days of establishing which resulted in the cultures developing myofibers and producing additional myosin heavy chain protein [42]. Also it appears that MGF-24aa-E significantly delays the entry of cells into senescence and increases moderately the proliferative life span of satellite cells isolated from neonatal and young but not from old adult skeletal muscle. These experiments allow the distinction between two specific roles of MGF E-24aa peptide and its effects on proliferation and differentiation that are required for the maintenance and then repair of the musculature throughout life (see Figures 4 and 5).

## 3. Conclusions

It has been known for some time that during aging there is a marked decline in muscle mass and strength which can be only partly ameliorated by continuing with resistance type of training. Exercise is to be encouraged as it has health benefits and a feeling of well-being during the aging process. However, in old age many individuals will become partially or only partially immobile. In the aging population day- to-day care will increase as a percentage of the elderly, and this will result in an ever-increasing financial commitment particularly in the advanced nations. For this and other reasons the large pharmaceutical companies have recently taken a serious interest in possible ways to increase muscle mass and strength in the elderly and in the many disease states that are accompanied by muscle loss. Much of their recent research is kept confidential for obvious reasons but can be appreciated somewhat from filed patents some of which had been filed by academic institutions before the present financial crisis. However, it seems that in the reasonably near future, when we understand better the signaling and the role of IGF-I growth factors, it should be possible to strengthen the musculature in the infirm so they can continue to carry out routine physical tasks. It has been known for some time that GH levels markedly decrease during aging and GH supplementation increases muscle mass in the elderly [28]. Serum IGF-I levels also decrease during aging but administration of IGF-I is problematic because of its insulin-like effect on blood sugar levels, and the main consensus in a point:counterpoint discussion was that IGF is not a major regulator of muscle mass [43]. Over the past decade or so, studies have demonstrated alternative splicing events occur in the IGF-1 gene in skeletal muscle in response to mechanical strain. This splicing results in the production of different 3′ mRNA and a different terminal amino acid sequence in main splice variants and isoforms. Initially there was confusion as the splice variants previously named IGF-IEb in the rat but IGF-IEc in the human are essentially the same although shift in the reading frame shift occurs at a slightly different place and the C terminal peptides (E domain) have just a few amino acids difference. In animals and humans this unique E domain has special functions. Because of this confusion the IGF-I splice variants were named mechano growth factor (MGF) as the splicing of the IGF-I gene responses to mechanical signals [21–28]. Experiments using a synthetic peptide of just 24 amino acids increased murine myoblast C2C12 cell cultures, and using myoblasts transfected with MGF cDNA showed proliferation in these mouse muscle cell cultures [24]. Some groups found the experiments with the E peptide difficult to repeat as unfortunately they used commercially available murine C2C12 cells which had been grown up so many times and very few, if any of these transformed and mutated cells behaved as muscle progenitor cells. Heinemeier et al. cloned the few progenitor cells by separating them from the transformed cells that continually multiplied whether or not they were exposed to MGF or IGF-I [44]. There was a lesson to be learnt from this, and the author's group concentrated on using human muscle mononucleated myoblasts obtained by biopsies from patients with muscle wasting diseases. It was that a 24 h treatment with this peptide increased the number of progenitor cells observed in primary cultures from healthy, ALS, and dystrophic human cells in vitro [41]. As far as aging is concerned our studies have shown that older muscles are less able to produce MGF, and this seemingly results in an age-related loss in both muscle mass and strength. Recent studies by Gillian Butler-Browne's group in Paris and our group in London who used primary cultures found that human mononucleated/desmin-expressing myoblasts [42] from young muscle biopsies reacted somewhat differently to those from elderly muscle when cultured. Muscle progenitor cells like other diploid somatic cells have a finite potential to divide before they become senescent but it was found that the myoblasts from older individuals still responded to the unique 24aa MGF peptide but in a somewhat different way. Treatment with the MGF-24aa-E peptide increased the proliferative capacity of the human myoblasts isolated from neonatal and young adult muscle. Those cultured from old adult muscle showed a marked increase in nuclei per myotube (see Figure 5). In the old muscle there are considerable numbers of quiescent “reserve” cells, and their activation by the unique E domain peptide increased in the number of nuclei in the myotubes. The primary action of the MGF E-24aa peptide on elderly muscle was to push reserve myoblasts through the next step of fusion to start the hypertrophy process. 

It seems that as we grow older the system becomes less sensitive for detecting mechanical strain because of the decreased compliance of the tissue due to cross-linking in the connective tissues. There is some evidence that the compliance of the muscle connective tissue is increased more than in sedentary individuals and this helps to maintain power output as well as fatigability. Recent research has shown that there are mechanosensor molecules such as focal adhesion kinase systems. These signalling molecules are wishbone in structure and open out when stretched. As their name denotes these FAKs are believed to involve activation of phosphatases which are possible part of the upstream activation of the splicing of the IGF-I gene to produce MGF. It has been shown that the signalling associated with MGF is different to other splice variants of the IGF-I gene from the human exercise studies carried out at McMaster University in Canada. The temporal response of MGF to acute damaging exercise results in *Myf5* and *MyoD* expression and the later response to IGF-IEa and IGF-I Eb to MRF-4 to the acute damaging repair phase [45] which occurs after the increase in the number of extra nuclei triggered by MGF (human IGF-1Ec). Confusion has arisen in this field of study because of the temptation to use murine myoblast cell lines such as C2C12 cells. These transformed, mutated cells that readily multiply are no longer suitable for this kind of study, and this message has not yet been appreciated by pharmaceutical companies or academic research groups. 

The question remains, why does not exercise produce as much MGF and the same muscle gain in the elderly as in young individuals? We do need to understand the age-related amelioration in the upstream signalling at the cellular and molecular levels as this apparently holds the clues as to why loss of muscle mass and strength and to put this in the context of the musculoskeletal system as a whole. Eccentric exercise has been found to be associated with Akt/mTor/p70 signalling [46] but we still do not know why there is no apparent relationship in young individuals and rate of stretch in eccentric contractions, and at present this is presumed to be the case in older individuals. Second messengers relating to MGF expression have also been studied by the team in the Bach Institute of the Russian Academy of Sciences, and protein kinases A and C and cAMP appear to be involved in MGF upregulation [47] and the specific type of exercise that should be more beneficial for maintaining musculature in the elderly [48]. MGF is expressed in other tissues following mechanical strain and tissue damage [49] and enhances myogenic precursor cell transplantation success [50]. It is expressed in tendons following mechanical stress and increased osteoblasts in bone after damage [44]. There are also beneficial effects on the repair of the myocardium [51–54]. MGF has been found to be very neuroprotective [55], and this includes protection against oxygen-free radical damage [53], and on this basis other aspects continuing to exercise as we get older can be recommended. Therefore, it seems that the splicing and expression of the IGF-I gene and its isoforms is central to our understanding of the progressive inability of tissue repair, and there are broader aspects then just decline in skeletal muscle function [54, 56]. Although the stabilized MGF E 244aa peptide can be purchased over the internet it is expensive, and apart from research it is apparently being used by body builders and doping for improved athletic performance. It is predicted that its use will be more widespread as it can be produced using recombinant methods in the same way as human insulin. A group of Russian scientists [57, 58] are producing a MGF peptide in this way and using it for bona fide reasons that include alcoholic myopathy and muscle atrophy resulting from space travel. There is also much interest in China where recombinant MGF is also being produced [59] but less is known about its uses in these countries but it is clear that they appreciate its potential for treating in tissue atrophy and repair.
